# Childhood Exposure to Interparental Physical Violence and Adult Cardiovascular Disease

**DOI:** 10.1001/jamanetworkopen.2024.51806

**Published:** 2024-12-20

**Authors:** Cancan Cui, Lin Liu, Haibin Li, Yitian Qi, Jiayin Song, Ning Han, Zhijia Wang, Xinyun Shang, Chen Sheng, Lois Balmer, Zhiyuan Wu

**Affiliations:** 1 Department of Radiology, China-Japan Union Hospital of Jilin University, Jilin University, Jilin, China; 2 Department of Cardiac Surgery, Beijing Chaoyang Hospital, Capital Medical University, Beijing, China; 3 Department of Nutrition, Harvard T.H. Chan School of Public Health, Boston, Massachusetts; 4 Centre for Precision Health, School of Medical and Health Sciences, Edith Cowan University, Perth, Australia

## Abstract

**Importance:**

Childhood adverse experiences have been linked with long-term risk of cardiovascular disease (CVD), yet the transgenerational associations between interparental behaviors and CVD remain poorly understood.

**Objectives:**

To explore the association between exposure to childhood interparental physical violence and the subsequent risk of CVD and to examine whether the association is modified by adult depressive symptoms.

**Design, Setting, and Participants:**

This population-based cohort study included data from the China Health and Retirement Longitudinal Study (CHARLS), an ongoing study recruiting individuals aged 45 years or older, dated between June 1, 2011, and December 31, 2020, with a follow-up duration of 9 years. The data were analyzed from October 1, 2023, to May 10, 2024.

**Exposures:**

An early life exposure questionnaire with information on the frequency of witnessing interparental physical violence was administered. Depressive symptoms were assessed via the validated 10-item Center for Epidemiologic Studies Depression Scale.

**Main Outcomes and Measures:**

The outcome measures included self-reported physician-diagnosed heart disease (defined as myocardial infarction, angina, coronary heart disease, heart failure, or other heart problems) and stroke. Multivariate Cox proportional hazards regression models using attained age as the time scale were conducted.

**Results:**

Of 10 424 participants, the mean (SD) age was 58.1 (9.0) years, 5332 (51.2%) were female, and 872 (8.4%) reported exposure to interparental physical violence. Exposure to childhood interparental physical violence was associated with increased risks of adult-onset CVD (hazard ratio [HR], 1.36; 95% CI, 1.20-1.55), heart disease (HR, 1.36; 95% CI, 1.17-1.57), and stroke (HR, 1.28; 95% CI, 1.03-1.61). Participants exposed to childhood interparental physical violence had a greater prevalence of depressive symptoms (2371 of 9335 participants [25.4%]), which mediated 11.0% of the association between childhood interparental physical violence and CVD (HR, 1.26; 95% CI, 1.09-1.45).

**Conclusions and Relevance:**

In this cohort study, childhood exposure to interparental physical violence was associated with a higher risk of adult-onset CVD, which was partially mediated by adult depressive symptoms. The findings emphasize the need for comprehensive strategies and policy efforts that address the social determinants of interparental violence and provide household education opportunities.

## Key Points

**Question:**

What is the association of childhood exposure to interparental physical violence with adult-onset cardiovascular disease (CVD)?

**Findings:**

In this cohort study of 10 424 participants, people who reported exposure to childhood interparental physical violence had a 36% greater risk of adult-onset CVD. The participants exposed to childhood interparental physical violence had a greater prevalence of depressive symptoms, which mediated 11% of the association between childhood interparental physical violence and CVD.

**Meaning:**

These findings suggest that exposure to interparental physical violence in early life is associated with the incidence of CVD in adulthood.

## Teaser

This cohort study explores the association between exposure to childhood interparental physical violence and the subsequent risk of adult-onset cardiovascular disease and examines whether the association is modified by adult depressive symptoms.

## Introduction

Early life adversities refer to a wide range of stressful experiences during childhood and adolescence, covering abuse, neglect, household challenges, and community-level stressors.^1,2,3^ Early life adversity remains a major public health and social welfare problem, given that a large proportion of children are exposed to adversities, according to the Behavioral Risk Factor Surveillance System.^4,5,6^ Adverse childhood experiences have gradually been recognized as social determinants of adult physical health, including cardiovascular disease (CVD).^7^

Recent population studies highlighted the associations between adverse childhood experiences, commonly classified into abuse (psychological, physical, or sexual), household dysfunction (eg, substance use by household members, mental illness, parental separation), and neglect with a range of cardiovascular risk factors predictive of subsequent cardiovascular events.^3,8,9,10,11,12^ The American Heart Association has issued health and policy statements highlighting the association of overall adverse childhood experiences with cardiometabolic health throughout the life course and suggesting potential pathways linking adverse childhood experiences and CVD by affecting health behaviors, pathophysiological factors, the immune system, psychological factors, and metabolic health integrity.^13,14,15^ Experiencing sexual abuse in early life was associated with a greater risk of cardiovascular mortality in adulthood.^16,17,18,19^ Moreover, parental behavioral indicators have been defined as adverse childhood experiences that underscore transgenerational effects (eg, parental incarceration and interparental violence).^20,21^ Exposure to parental incarceration has been linked with CVD through stressors on neurobiological systems.^22^ However, few population studies to date have explored the proportions of community-based populations exposed to childhood interparental physical violence and the subsequent associations with adult-onset CVD.

Therefore, we examined the associations of childhood exposure to interparental physical violence with the subsequent risk of CVD onset. We also explored the potential mediating role of adult depressive symptoms in a nationally representative cohort.

## Methods

### Setting and Data Source

The China Health and Retirement Longitudinal Study (CHARLS) is an ongoing prospective cohort established in 2011 by recruiting adults aged 45 years or older. The participants were followed biennially thereafter via face-to-face questionnaires and in-person physical examinations. This study includes data from June 1, 2011, to December 31, 2020. Details of the study profile have been previously published.^23,24^ The protocols of CHARLS were approved by the Ethics Review Committee of Peking University. All participants provided written informed consent before participation. The study followed the Strengthening the Reporting of Observational Studies in Epidemiology (STROBE) reporting guidelines.

In this study, our analyses were restricted to individuals who participated in the 2011 survey as a baseline and the early life experience survey in 2014. Participants were excluded if they had missing data on age or sex; lacked data on childhood exposure to interparental violence; had a history of cancer or CVD; or were lost to follow-up during the subsequent biannual follow-up in 2013, 2015, 2018, and 2020. A detailed flowchart of the inclusion and exclusion procedures is presented in the eFigure in Supplement 1.

### Definition of Childhood Interparental Physical Violence

Information on early life adversity experiences, defined as childhood adversities before 17 years of age, was collected through face-to-face interviews during the 2014 life history survey.^25^ In detail, participants were asked “[Has] your father ever beat up your mother? Or [has] your mother ever beat up your father?” The response options for each item included never, not very often, sometimes, and often. Participants who responded sometimes or often to either of these 2 questions were defined as the exposure group.^25^ The frequency of interparental physical violence was also analyzed.

### Determination of CVD

In accordance with previous studies,^26^ the primary outcome was defined as nonfatal CVD events confirmed by the following questions: “Have you been told by a doctor that you have been diagnosed with a heart attack, angina, coronary heart disease, heart failure, or other heart problems?” or “Have you been told by a doctor that you have been diagnosed with a stroke?” Participants who responded affirmatively to either of the 2 questions during follow-up surveys were defined as having incident CVD. The outcomes were assessed by trained interviewers who are harmonized to international leading aging surveys in the Health and Retirement Study.^27^ Data on cardiovascular death were not available for the CHARLS cohort.

### Assessment of Covariates

Demographic and health-related data were collected through face-to-face interviews. Demographic information included age, sex (male or female), residential area (rural or urban), marital status (married or other), and educational level (primary school or below, middle school or above). Health behavior factors included current smoking and current alcohol consumption. Body mass index (BMI) was calculated as self-reported weight in kilograms divided by height in meters squared. Health status factors included self-reported physician-diagnosed comorbidities (hypertension, diabetes, dyslipidemia) and the use of medications for hypertension, diabetes, and dyslipidemia. Childhood physical abuse by parents was asked, “When you were growing up, did your female/male guardian ever hit you?”^25^ Depressive symptoms were measured via the short form of the 10-item Center for Epidemiologic Studies Depression Scale. A total score of 12 or higher was used to indicate depressive symptoms.^28,29^ Details are shown in the eMethods in Supplement 1.

### Statistical Analysis

The data were analyzed from October 1, 2023, to May 10, 2024. Baseline variables are presented as mean (SD) values for continuous variables, and proportions and percentages are presented for categorical variables. There were 3.1% (319 of 10 424) of the total data items missing for the covariates of residence, educational level, smoking status, and current drinking status. In the main analysis, multiple imputation by chained equations was applied to address the missing data associated with the covariates.

We computed the person-time of follow-up for each participant from baseline to the follow-up date of CVD diagnosis, death, or the end of follow-up, whichever came first. The associations of exposure to childhood interparental violence and the frequency of interparental violence experience with incident CVD were assessed using multivariable Cox proportional hazards regression models and are presented as hazard ratios (HRs) with 95% CIs. Attained age at baseline and at the end of follow-up was used as the time scale for the Cox proportional hazards regression analyses.^30^ The proportional hazard assumption was evaluated using Schoenfeld residuals. We used the Bonferroni correction method to account for false positives (*P* < .05 / 3 = .017 for CVD, heart disease [defined as myocardial infarction, angina, coronary heart disease, heart failure, or other heart problems], and stroke). To account for confounding factors, we conducted 2 models: age and sex were adjusted in model 1; and age, sex, residence, educational level, smoking status, and current drinking status were adjusted in model 2. Given that BMI status and the comorbidities of hypertension, dyslipidemia, and diabetes could be potential mediators, these variables were not adjusted for in the main analysis.^15,19^ We conducted a series of sensitivity analyses to assess the missing bias (repeating the analysis among the complete data of 10 105 participants); further adjust for BMI status, hypertension, dyslipidemia, diabetes, and medication use (antihypertensive, antidiabetic, lipid lowering); apply the inverse probability of treatment weighting (IPTW) method to further control for confounding bias; and consider death as the competing event using Fine-Gray subdistribution models. Considering the potential interaction,^31^ we analyzed the associations between childhood exposure to interparental violence and CVD stratified by sex, childhood physical abuse by parent, and adult depressive symptoms. We tested the multiplicative interaction by adding the product term to the Cox proportional hazards regression model.

To evaluate the population-level association of childhood exposure to interparental violence with the risk of CVD, we calculated the time-dependent population attributable fraction (PAF) and compared the magnitudes between interparental violence and depressive symptoms. To investigate whether the association of childhood exposure to interparental violence with CVD was modified by the presence of adult depressive symptoms, we conducted a mediation analysis by calculating the percentage of excess risk mediated to represent the mediation proportions.^32^ A detailed description of the methods is provided in the eMethods in Supplement 1.

All statistical analyses were performed using R software, version 4.2.1 (R Project for Statistical Computing). A 2-sided *P* < .05 was considered statistically significant.

## Results

### Participant Characteristics

Table 1 shows the baseline characteristics according to the frequency of experiencing childhood interparental physical violence. Of 10 424 participants, the mean (SD) age was 58.1 (9.0) years, 5332 (51.2%) were female, and 5092 (48.8%) were male. Among the total participants, 872 (8.4%) reported childhood exposure to interparental physical violence. Compared with those who never experienced interparental physical violence, participants who were often exposed in childhood to interparental violence were more likely to achieve lower educational levels, experience childhood physical abuse by parents, and have depressive symptoms as adults (Table 1).

**Table 1.** Baseline Characteristics of Participants by Childhood Exposure to Interparental Violence

Characteristic^a^	Overall (N = 10 424)	Frequency of interparental physical violence^b^			
		Never (n = 8244)	Rarely (n = 1308)	Sometimes (n = 685)	Often (n = 187)
Age, mean (SD), y	58.1 (9.0)	58.1 (9.0)	57.8 (9.1)	57.9 (8.8)	58.0 (8.7)
Male, No. (%)	5092 (48.8)	3937 (47.8)	760 (58.1)	330 (48.2)	65 (34.8)
Female, No. (%)	5332 (51.2)	4307 (52.2)	548 (41.9)	355 (51.8)	122 (65.2)
Rural residence, No. (%)	8540 (82.0)	6694 (81.3)	1104 (84.6)	587 (85.7)	155 (82.9)
Married, No. (%)	9378 (90.0)	7408 (89.9)	1185 (90.6)	615 (89.8)	170 (90.9)
Educational level, No. (%)					
Primary school or below	6866 (65.9)	5408 (65.7)	859 (65.7)	460 (67.2)	139 (74.3)
Middle school or above	3550 (34.1)	2829 (34.3)	448 (34.3)	225 (32.8)	48 (25.7)
Current smoking, No. (%)	3132 (30.0)	2430 (29.5)	445 (34.0)	227 (33.1)	30 (16.0)
Current drinking, No. (%)	3677 (35.3)	2822 (34.3)	546 (41.8)	257 (37.6)	52 (27.8)
BMI, mean (SD)	23.4 (3.8)	23.5 (3.9)	23.1 (3.4)	23.3 (3.6)	23.5 (3.7)
Obesity, No. (%)^c^	888 (8.5)	737 (8.9)	84 (6.1)	51 (7.4)	16 (8.6)
Comorbidity, No. (%)					
Hypertension	2077 (19.9)	1680 (20.4)	219 (16.7)	143 (20.9)	35 (18.7)
Diabetes	470 (4.5)	370 (4.5)	49 (3.7)	37 (5.4)	14 (7.5)
Dyslipidemia	718 (6.9)	574 (7.0)	72 (5.5)	61 (8.9)	11 (5.9)
Medication use, No. (%)					
Antihypertensive	1461 (14.0)	1207 (14.6)	138 (10.6)	93 (13.6)	23 (12.3)
Antidiabetic	300 (2.9)	240 (2.9)	34 (2.6)	18 (2.6)	8 (4.3)
Lipid lowering	336 (3.2)	265 (3.2)	38 (2.9)	28 (4.1)	5 (2.7)
Depressive symptoms, No. (%)^d^	2371 (22.7)	1766 (23.8)	346 (26.5)	204 (29.8)	55 (29.4)
Childhood physical abuse by parents, No. (%)	2985 (29.1)	1992 (21.4)	470 (36.3)	410 (60.5)	113 (60.4)

Abbreviation: BMI, body mass index (calculated using self-reported weight in kilograms divided by height in meters squared).

^a^ Covariate missingness: residence (n = 9), educational level (n = 8), current smoking (n = 305), current drinking (n = 9), BMI (n = 1887), and depressive symptoms (n = 1089).

^b^ Childhood exposure to interparental violence was defined as witnessing interparental violence before participants were 17 years of age.

^c^ Obesity was defined as a BMI of 28.0 or more for the Chinese population.

^d^ Depressive symptoms were defined as a 10-item Center for Epidemiologic Studies Depression Scale score of 12 or more.

### Interparental Physical Violence and CVD

**Figure 1.**
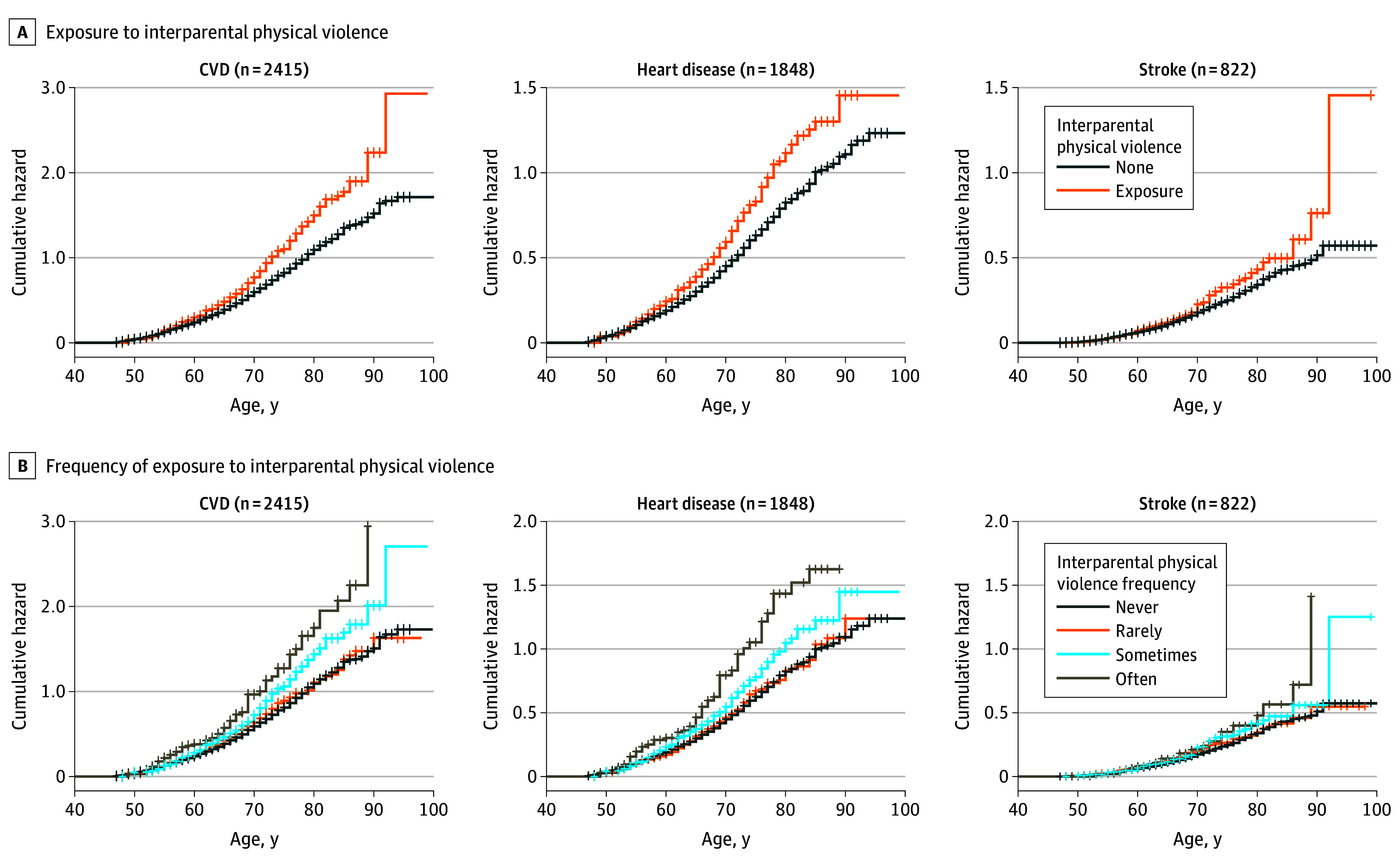
Cumulative Hazard of Cardiovascular Disease (CVD) According to Interparental Physical Violence Experience Heart disease included myocardial infarction, angina, coronary heart disease, heart failure, or other heart problems. Childhood exposure to interparental violence was defined as witnessing interparental violence sometimes or often before participants were 17 years of age.

During a maximum follow-up of 9 years, there were 2415 cases (23.2%) of physician-diagnosed CVD, including 1848 incident cases (17.7%) of heart disease and 822 incident cases (7.9%) of stroke. We found that people exposed to interparental physical violence during childhood had a higher cumulative incidence of CVD than did those without such experience (38.9 vs 29.0 per 1000 person-years) (Table 2) and cumulative hazard as shown in Figure 1. In the multivariable adjusted model, the HRs corresponding to childhood exposure to interparental physical violence were 1.36 (95% CI, 1.20-1.55) for total CVD, 1.36 (95% CI, 1.17-1.57) for heart disease, and 1.28 (95% CI, 1.03-1.61) for stroke (Table 2). When we analyzed the frequency of experiencing childhood interparental physical violence, we observed significant trends indicating that more frequent exposure to interparental physical violence was associated with an increased risk of CVD (sometimes: HR, 1.32 [95% CI, 1.14-1.53]; often: HR, 1.59 [95% CI, 1.24-2.04]; *P* < .001 for trend), heart disease (sometimes: HR, 1.30 [95% CI, 1.10-1.54]; often: HR, 1.63 [95% CI, 1.23-2.16]; *P* < .001 for trend), and stroke (sometimes: HR, 1.24 [95% CI, 0.96-1.60]; often: HR, 1.52 [0.98-2.35]; *P* = .02 for trend) (Table 3).

**Table 2.** Association Between Childhood Exposure to Interparental Physical Violence and Incident CVD, 2011-2020

Outcome	No. (incidence rate per 1000 person-years)	Model 1^a^		Model 2^b^	
		HR (95% CI)	*P* value	HR (95% CI)	*P* value
CVD					
No interparental violence	2157 (29.0)	1 [Reference]	NA	NA	NA
Interparental violence^c^	258 (38.9)	1.35 (1.19-1.54)	<.001	1.36 (1.20-1.55)	<.001
Heart disease^d^					
No interparental violence	1650 (21.8)	1 [Reference]	NA	NA	NA
Interparental violence	198 (29.4)	1.34 (1.16-1.56)	<.001	1.36 (1.17-1.57)	<.001
Stroke					
No interparental violence	737 (9.4)	1 [Reference]	NA	NA	NA
Interparental violence	85 (11.9)	1.28 (1.02-1.60)	.03	1.28 (1.03-1.61)	.03

Abbreviations: CVD, cardiovascular disease; HR, hazard ratio; NA, not applicable.

^a^ Model 1: adjusted for age and sex.

^b^ Model 2: adjusted for age, sex, residence, educational level, smoking status, and current drinking status.

^c^ Childhood exposure to interparental violence was defined as witnessing interparental violence sometimes or often when participants were younger than 17 years.

^d^ Heart disease was defined as myocardial infarction, angina, coronary heart disease, heart failure, or other heart problems.

**Table 3.** Association Between the Frequency of Witnessing Interparental Physical Violence and Incident CVD, 2011-2020

Frequency of witnessing interparental physical violence	No. (incidence rate per 1000 person-years)	Model 1^a^		Model 2^b^	
		HR (95% CI)	*P* value	HR (95% CI)	*P* value
Total CVD					
None	1853 (28.8)	1 [Reference]	NA	NA	NA
Rarely	304 (29.9)	1.07 (0.95-1.21)	.26	1.09 (0.96-1.23)	.19
Sometimes	194 (37.0)	1.31 (1.13-1.51)	<.001	1.32 (1.14-1.53)	<.001
Often	64 (46.0)	1.58 (1.23-2.03)	<.001	1.59 (1.24-2.04)	<.001
*P* value for trend^c^	NA	NA	<.001	NA	<.001
Heart disease^d^					
None	1420 (21.8)	1 [Reference]	NA	NA	NA
Rarely	230 (22.2)	1.07 (0.93-1.23)	.38	1.08 (0.94-1.24)	.28
Sometimes	147 (27.6)	1.28 (1.08-1.52)	.004	1.30 (1.10-1.54)	.002
Often	51 (36.3)	1.63 (1.23-2.15)	.001	1.63 (1.23-2.16)	.001
*P* value for trend	NA	NA	<.001	NA	<.001
Stroke					
None	631 (9.3)	1 [Reference]	NA	NA	NA
Rarely	106 (9.9)	1.07 (0.87-1.32)	.51	1.08 (0.88-1.33)	.48
Sometimes	64 (11.3)	1.23 (0.95-1.59)	.12	1.24 (0.96-1.60)	.10
Often	21 (13.8)	1.53 (0.99-2.36)	.06	1.52 (0.98-2.35)	.06
*P* value for trend	NA	NA	.02	NA	.02

Abbreviations: CVD, cardiovascular disease; HR, hazard ratio; NA, not applicable.

^a^ Model 1: adjusted for age and sex.

^b^ Model 2: adjusted for age, sex, residence, educational level, smoking status, and current drinking status.

^c^ *P* value for trend was calculated using the frequency of witnessing interparental violence as a continuous variable.

^d^ Heart disease included myocardial infarction, angina, coronary heart disease, heart failure, or other heart problems.

### Sensitivity Analysis and Subgroup Analysis

The associations between childhood exposure to interparental physical violence and adult-onset CVD were largely consistent after restricting the analysis among the complete data; additionally adjusting for BMI status, comorbidity, and medication use; applying the IPTW method; and considering the competing risk of death. eTable 1 in Supplement 1 summarizes the baseline characteristics between the whole dataset and complete dataset (n = 10 105), suggesting that there was no statistically significant difference. Similar results were observed when additional adjustments were performed, and IPTW was used to further account for confounding (eTable 2 in Supplement 1). Considering the competing risk of death, the HRs were slightly attenuated but still remained significant (eTable 3 in Supplement 1). There was no significant interaction between childhood exposure to interparental physical violence and sex, childhood physical abuse, or adult depressive symptoms and the risk of CVD, heart disease, or stroke (all *P* > .10 for interaction) (eTable 4 in Supplement 1). The HRs corresponding to childhood exposure to interparental physical violence were 1.37 (1.12-1.67) for males and 1.35 (1.14-1.60) for females (*P* = .88 for interaction).

### PAF Associated With Childhood Interparental Physical Violence

**Figure 2.**
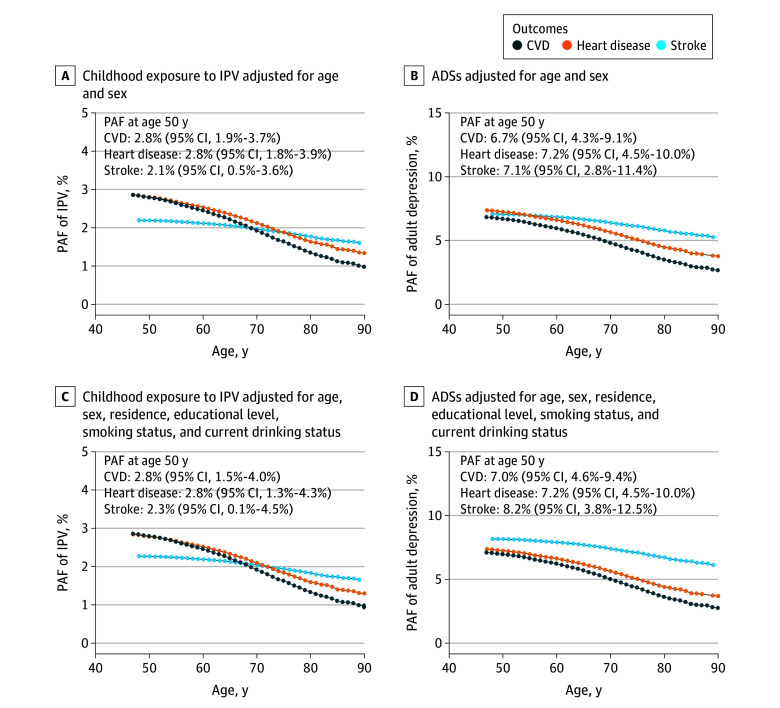
Age-Dependent Population Attributable Fraction (PAF) of Childhood Exposure to Interparental Physical Violence (IPV) and Adult Depressive Symptoms (ADSs) of Cardiovascular Disease (CVD) Heart disease included myocardial infarction, angina, coronary heart disease, heart failure, or other heart problems. Childhood exposure to IPV was defined as witnessing IPV sometimes or often before participants were 17 years of age.

Figure 2 presents the age-dependent PAFs of CVD, heart disease, and stroke associated with childhood exposure to interparental physical violence and adult depressive symptoms. According to the adjusted model, the PAFs at age 50 years associated with childhood exposure to interparental physical violence were 2.8% (95% CI, 1.5%-4.0%) for CVD, 2.8% (95% CI, 1.3%-4.3%) for heart disease, and 2.3% (95% CI, 0.1%-4.5%) for stroke. The PAFs at age 50 years of adult depressive symptoms were 7.0% (95% CI, 4.6%-9.4%) for CVD, 7.2% (95% CI, 4.5%-10.0%) for heart disease, and 8.2% (95% CI, 3.8%-12.5%) for stroke.

### Mediation of Adult Depressive Symptoms

At baseline, 2371 of the 9335 participants (25.4%) with available data on the 10-item Center for Epidemiologic Studies Depression Scale questionnaire had depressive symptoms. We found that childhood exposure to interparental physical violence was significantly associated with adult depressive symptoms (eTable 5 in Supplement 1). In the fully adjusted model, the percentage of excess risk mediated was 11.0% for total CVD (HR, 1.26; 95% CI, 1.09-1.45), 11.1% for heart disease (HR, 1.27; 95% CI, 1.08-1.49), and 15.6% for stroke (HR, 1.17; 95% CI, 0.94-1.50), although the association between childhood exposure to interparental violence and stroke was attenuated to insignificance after adjusting for depressive symptoms (eTable 6 in Supplement 1).

## Discussion

In this nationally representative cohort study, we found increased CVD risk among adults who were exposed to childhood interparental physical violence. The PAF suggested that approximately 2.8% of the risk of CVD could be associated with childhood exposure to interparental violence if this was a causal relationship. There was no significant interaction between childhood interparental physical violence and adult depressive symptoms, whereas 11.0% of the association between childhood interparental violence and CVD was mediated by depressive symptoms. Our findings underscore the importance of addressing the problem of household violence in early life as a significant public health issue.

Previous studies reported that overall adverse childhood experiences, including some domains of family violence, such as physical abuse, were associated with a greater risk of CVD and a range of cardiovascular risk factors.^3,17,19,33,34,35^ In the UK Biobank, Ho and colleagues^36^ found that one-third of participants reported at least one type of child maltreatment, and there was a dose-response relationship between the number of maltreatment types and incident CVD. However, few population studies to date have explored the association of childhood exposure to interparental violence with the subsequent long-term risk of CVD. Lin and colleagues^19^ reported a greater risk of 14 chronic diseases and multimorbidity associated with 12 adverse childhood experiences using a cross-sectional analysis, which included some domains of family violence.

Our study extends the existing evidence regarding early life household violence and CVD risk. We included a national prospective cohort with information on childhood interparental violence experience, allowing us to investigate the associations of exposure and frequency of childhood interparental violence experience with CVD in adults. We found that childhood exposure to interparental physical violence was associated with a greater risk of CVD in later life, and there was a significant trend toward increasing CVD risk with increasing frequency of experiencing interparental violence. The associations between childhood exposure to interparental violence and CVD onset were similar among males and females but seemed to be stronger for heart disease among females and predominant for stroke among males. A previous study reported that childhood maltreatment was associated with heart disease, with stronger associations observed among females.^37^ However, the results from a cross-sectional analysis^19^ revealed that the odds ratio of stroke was not statistically significant when participants with 4 or more adverse childhood events and those with no adverse childhood events were compared. Consistent results were observed among the UK Biobank samples.^37^

The transgenerational effects of parental behaviors on the next generation have attracted increased awareness in recent decades. Lee et al^38^ reported that parental incarceration was associated with higher risk of myocardial infarction and stroke among women but not men. The US National Longitudinal Study of Adolescent to Adult Health reported that childhood parental incarceration was associated with 33% higher adjusted odds of developing adult-onset hypertension and 60% higher adjusted odds of developing adult-onset inflammation, highlighting the possible transgenerational health consequences of incarceration and parental misbehaviors.^22^ Mental health consequences have also been implicated.^38^ Our analysis revealed that 8.4% of the participants reported exposure to interparental physical violence during childhood. The PAF of CVD associated with childhood exposure to interparental violence was 2.8%. These findings increase awareness of the transgenerational effects of interparental violence and highlight the need to reduce household violence.

The exact mechanism underlying the associations of childhood adversity and interparental violence with incident CVD remains unclear. Reviews have suggested that exposure to interparental violence may increase CVD through behavioral, mental, biological, social, and cognitive pathways. Participants who experienced early life adversity are at a higher risk of alcohol abuse, smoking, poor diet, and other risk behaviors but have poor educational attainment,^39,40,41^ which are important social and behavioral determinants of cardiovascular health. In addition, childhood adversity is closely associated with biological biomarkers (such as inflammation, lipids, and biological aging) and metabolic health status, which could lead to CVD.^15,19,42,43,44^ Prolonged or excessive activation of allostatic pathways could also be involved in the association of childhood adversity or family issues with health.^45^ Studies have suggested that the association between childhood adversity and CVD is mediated by psychological risk factors.^36,46,47^ Our study suggests that exposure to interparental violence was associated with incident CVD, partially through adult depressive symptoms, which is consistent with the findings of previous studies.^18,33,34,36,43^

Given the burdens and adverse health consequences of childhood exposure to interparental violence documented in the literature and our study, public health interventions are dedicated to the prevention of household violence issues.^4^ In a systematic review, millions of adults across Europe and North America faced early life adversity, and a 10% reduction in childhood adversity prevalence could equate to annual savings of 3 million disability-adjusted life-years or $105 billion.^47^ Therefore, increasing expenditures to ensure a safe and nurturing household environment through public education would relieve pressure on health care systems.^48^ In addition, lifestyle and mental health should be emphasized and targeted among those exposed to interparental violence or other household violence to mitigate deleterious health outcomes.^36,49^

### Strengths and Limitations

This study has some strengths, including the large sample used to explore the associations between childhood exposure to interparental physical violence and adult CVD. We also compared the PAFs of childhood exposure to interparental violence and explored whether adult depressive symptoms mediated the association with CVD.

This study also has some limitations. First, data on covariates and depressive symptoms were available for only a proportion of participants; thus, the study findings should be interpreted with caution when referring to these covariates. However, significant associations between childhood exposure to interparental violence and CVD remained after the analysis of the whole dataset was repeated without missing data, suggesting the robustness of the study’s findings. Second, data on childhood interparental violence experience were collected retrospectively and were subject to recall bias. These response options were subjective, and people who experienced severe childhood interparental physical violence may be unwilling to report their exact adverse experiences. However, a previous study has shown that retrospective measurements of childhood adversity experiences have good test-retest reliability and may provide some unique and complementary information compared with prospective measures.^50^ Previous studies also revealed significant associations between childhood interparental physical violence and health outcomes, even though the effect size may be underestimated, supporting the association between childhood exposure to interparental physical violence and CVD in our analysis.^51^ Third, the associations of childhood interparental violence at different ages with CVD may differ, and more evidence is needed to reveal the vulnerable life-course window to household violence. Fourth, it is uncertain how generalizable our findings might be to other populations because of differences in household culture and socioeconomic status.

## Conclusions

In this cohort study, we found that childhood exposure to interparental physical violence was significantly associated with a higher risk of adult-onset CVD. Adult depressive symptoms partially mediated the association between childhood exposure to interparental violence and incident CVD. The findings emphasize the need for comprehensive strategies and policy efforts that address the social determinants of interparental violence and provide household education opportunities.

## Notes

Supplement 1. **eMethods.** Definition of Depression, and Statistical Methods **eTable 1.** Comparison of Characteristics Between Original and Complete Data Sets **eTable 2.** Sensitivity Analysis of Interparental Physical Violence Experience and Incident Cardiovascular Disease, 2011-2020 **eTable 3.** Interparental Physical Violence Experience and Incident Cardiovascular Disease Considering the Competing Risk of Death **eTable 4.** Subgroup Associations of Interparental Physical Violence Experience and Incident Cardiovascular Disease **eTable 5.** Association of Interparental Physical Violence Experience and Adult Depressive Symptoms at Baseline, 2011 **eTable 6.** Proportions of the Association Between Childhood Interparental Physical Violence Exposure and Cardiovascular Diseases Attributable to Adult Depressive Symptoms **eFigure.** Inclusion and Exclusion Flowchart for Participants in the Study

Supplement 2.
Data Sharing Statement
