# Supplementary material for: Childhood Exposure to Interparental Physical Violence and Adult Cardiovascular Disease
Source: JAMA Netw Open. 2024 Dec 20;7(12):e2451806. doi: 10.1001/jamanetworkopen.2024.51806 (PMC11662254; doi:10.1001/jamanetworkopen.2024.51806)
Supplement: Supplement 1. — eMethods. Definition of Depression, and Statistical Methods eTable 1. Comparison of Characteristics Between Original and Complete Data Sets eTable 2. Sensitivity Analysis of Interparental Physical Violence Experience and Incident Cardiovascular Disease, 2011-2020 eTable 3. Interparental Physical Violence Experience and Incident Cardiovascular Disease Considering the Competing Risk of Death eTable 4. Subgroup Associations of Interparental Physical Violence Experience and Incident Cardiovascular Disease eTable 5. Association of Interparental Physical Violence Experience and Adult Depressive Symptoms at Baseline, 2011 eTable 6. Proportions of the Association Between Childhood Interparental Physical Violence Exposure and Cardiovascular Diseases Attributable to Adult Depressive Symptoms eFigure. Inclusion and Exclusion Flowchart for Participants in the Study [file jamanetwopen-e2451806-s001.pdf]

Supplementary Online Content

Cui C, Liu L, Li H, et al. Childhood exposure to interparental physical violence and adult cardiovascular disease. *JAMA Netw Open.* 2024;7(12):e2451806.
doi:10.1001/jamanetworkopen.2024.51806

eMethods. Definition of Depression, and Statistical Methods
eTable 1. Comparison of Characteristics Between Original and Complete Data Sets
eTable 2. Sensitivity Analysis of Interparental Physical Violence Experience and Incident Cardiovascular Disease, 2011-2020
eTable 3. Interparental Physical Violence Experience and Incident Cardiovascular Disease Considering the Competing Risk of Death
eTable 4. Subgroup Associations of Interparental Physical Violence Experience and Incident Cardiovascular Disease
eTable 5. Association of Interparental Physical Violence Experience and Adult Depressive Symptoms at Baseline, 2011
eTable 6. Proportions of the Association Between Childhood Interparental Physical Violence Exposure and Cardiovascular Diseases Attributable to Adult Depressive Symptoms
eFigure. Inclusion and Exclusion Flowchart for Participants in the Study

This supplementary material has been provided by the authors to give readers additional information about their work.

© 2024 Cui C et al. *JAMA Network Open.*

eMethods. Definition of Depression, and Statistical Methods

Definition of depressive symptoms

Depressive symptoms were measured using the CES-D short form consisting of 10 items: (1) bothered by little things, (2) had trouble concentrating, (3) felt depressed, (4) everything was an effort, (5) felt hopeful, (6) felt fearful, (7) sleep was restless, (8) felt happy, (9) felt lonely, and (10) could not get going. Response options for each depressive symptom were measured from 0 (rarely or none of the time in the past week) to 3 (most or all of the time in the past week) and summed up ranging from 0 to 30. Items 5 and 8 were reversely scored before summing up.

Statistical analysis

To estimate population-level risks of cardiovascular disease attributable to childhood interparental violence exposure and adult depression, we calculated the average population-attributable fractions (PAFs) and 95% CIs based on Cox proportional hazard regression with the function of AFcoxph using 'AF' R package. Consistent with the HR estimation, PAFs for childhood interparental violence exposure and adult depression were calculated separately using two models: age and sex were adjusted in model 1; age, sex, residence, marriage status, education level, smoking status, current drinking, and relationship with parents were adjusted in model 2. By including different possible orders by which the risk factors could be eliminated from the population, the average PAF estimates average the different attributable fractions from each permutation, which allows partitioning the overall risk into exposure-specific contributions and comparing different risk factors concerning their respective independent impact on the outcome of interest and prevalence of the risk factors. AFcoxph estimates the attributable fraction for a time-to-event outcome under the hypothetical scenario where a binary exposure X is eliminated from the population, developed by Sjolander and Vanstee-landt.

AF = 1 − {1 − S0(t)} {1 − S(t)}, where S0(t) denotes the counterfactual survival function for the event if the exposure would have been eliminated from the population at baseline and S(t) denotes the factual survival function. If Z is sufficient for confounding control, then S0(t) can be expressed as EZ{S(t | X = 0,Z)}. The function uses a fitted Cox proportional hazards regression to estimate S(t | X = 0, Z), and the marginal sample distribution of Z to approximate the outer expectation.

© 2024 Cui C et al. *JAMA Network Open.*

Percentage of excess risk mediated (PERM) is calculated as the proportion of the indirect effect mediated through the intermediate variable relative to the total effect of the exposure on the outcome. In our analysis, we calculated the PERM as:

PERM=(hazard ratio (age, sex, residence, education level, smoking status, current drinking)-hazard ratio (age, sex, residence, education level, smoking status, current drinking, depression adjusted))/(hazard ratio (age, sex, residence, education level, smoking status, current drinking)-1).

© 2024 Cui C et al. *JAMA Network Open.*

eTable 1. Comparison of Characteristics Between Original and Complete Data Sets

	Original	Complete [a]	*P* value [e]
Participants, No.	10424	10105	
Age, years	58.05(9.00)	58.10(8.99)	.68
Male, n (%)	5092(48.8)	4749(47.5)	.05
Female, n (%)	5332(51.2)	5308(52.5)	
Rural residence, n (%)	8540(82.0)	8278(81.9)	.90
Married, n (%)	9378(90.0)	9070(89.8)	.64
Education, n (%)			.51
primary or below	6866(65.9)	6706(66.4)	
middle school or above	3550(34.1)	3399(33.6)	
Current smoking, n (%)	3132(31.0)	3128(31.0)	>.99
Current drinking, n (%)	3677(35.3)	3474(34.4)	.17
BMI [b], kg/m^2	23.43(3.78)	23.43(3.78)	.94
Obesity [c], n (%)	888(10.4)	887(8.8)	.98
Comorbidity, n (%)			
hypertension	2077(19.9)	2019(20.0)	.94
diabetes	470(4.5)	459(4.5)	.94
dyslipidemia	718(6.9)	703(7.0)	.87
Medication use, n (%)			
antihypertensive	1461(14.0)	1419(14.0)	.97
antidiabetic	300(2.9)	290(2.9)	>.99
lipid lowering	336(3.2)	328(3.2)	.96
Depressive symptoms [d], n (%)	2371(22.7)	2371(23.5)	.97
Childhood physical abuse by parents, n (%)	2985(28.6)	2862(28.3)	.63

Data are presented as the mean (SD) or number (%), as appropriate.

a Complete data was defined as no missing data on covariates of residence, education, relationship with parents, smoking, alcohol.

b BMI was calculated using self-reported weight in kilograms divided by height in meters squared.

c Obesity was defined as BMI ≥28.0 kg/m^2 for Chinese population.

d Depressive symptoms was defined as CES-D score ≥12.

e *P* value based on ANOVA test or $\chi 2$ test as appropriate.

© 2024 Cui C et al. *JAMA Network Open.*

eTable 2. Sensitivity Analysis of Interparental Physical Violence Experience and Incident Cardiovascular Disease, 2011-2020

	Sensitivity 1 [d]		Sensitivity 2 [e]		Sensitivity 3 [f]	
	HR (95% CI)	P value	HR (95% CI)	P value	HR (95% CI)	P value
**Dichotomous**						
CVD						
No-exposure	Reference					
Exposure [a]	1.354(1.187-1.544)	<.001	1.341(1.178-1.527)	<.001	1.382(1.213-1.573)	<.001
Heart disease [b]						
No-exposure	Reference					
Exposure	1.36(1.171-1.579)	<.001	1.332(1.149-1.544)	<.001	1.371(1.182-1.591)	<.001
Stroke						
No-exposure	Reference					
Exposure	1.262(1.002-1.588)	.048	1.26(1.006-1.579)	.04	1.305(1.041-1.635)	.02
**Frequency**						
CVD						
None	Reference					
Rarely	1.091(0.964-1.235)	.17	1.132(1.002-1.279)	.046	1.087(0.961-1.23)	.18
Sometimes	1.316(1.132-1.53)	<.001	1.298(1.119-1.506)	.001	1.338(1.152-1.553)	<.001
Often	1.567(1.213-2.023)	.001	1.608(1.253-2.064)	<.001	1.531(1.169-2.005)	.002
P for trend [c]		<.001		<.001		<.001
Heart disease						
None	Reference					
Rarely	1.081(0.938-1.247)	.28	1.124(0.977-1.293)	.10	1.084(0.942-1.248)	.26
Sometimes	1.298(1.092-1.542)	.003	1.271(1.072-1.507)	.006	1.32(1.112-1.567)	.002
Often	1.654(1.247-2.194)	<.001	1.659(1.254-2.195)	<.001	1.515(1.119-2.051)	.007
P for trend		<.001		<.001		<.001
Stroke						
None	Reference					
Rarely	1.1(0.892-1.357)	.37	1.126(0.915-1.385)	.26	1.079(0.875-1.33)	.48
Sometimes	1.239(0.954-1.609)	.11	1.213(0.937-1.57)	.14	1.248(0.963-1.617)	.09
Often	1.428(0.904-2.257)	.13	1.543(0.998-2.387)	.05	1.611(1.005-2.583)	.048
P for trend		.03		.01		.02

Abbreviation: HR, hazard ratio; CI, confidence interval; CVD, cardiovascular disease

a Childhood interparental violence exposure was defined as witnessing interparental violence sometimes or often when participants were aged before 17 years.

b Heart disease included heart attack, angina, coronary heart disease, heart failure, or other heart problems.

c P for trend was calculated using frequency of witnessing interparental violence ranging between none and often as a continuous variable.

© 2024 Cui C et al. *JAMA Network Open.*

d Sensitivity 1: based on complete data (n=10 105) without missing for covariates. Adjusted for age, sex, residence, education, smoking status, and current drinking.

e Sensitivity 2: adjusted for age, sex, residence, education, smoking status, current drinking, obesity statue, hypertension, diabetes, dyslipidemia, and uses of medications.

f Sensitivity 3: age, sex, residence, education, smoking status, and current drinking were weighted by inverse probability of treatment weighting (IPTW).

© 2024 Cui C et al. *JAMA Network Open.*

eTable 3. Interparental Physical Violence Experience and Incident Cardiovascular Disease Considering the Competing Risk of Death

	Model 1 [d]		Model 2 [e]	
	HR (95% CI)	P value	HR (95% CI)	P value
CVD				
No-exposure	Reference			
Exposure [a]: Cox	1.352 (1.188-1.538)	<.001	1.364(1.199-1.552)	<.001
Exposure: Fine-Gray [b]	1.346(1.192-1.52)	<.001	1.357(1.201-1.532)	<.001
Heart disease [c]				
No-exposure	Reference			
Exposure: Cox	1.342 (1.158-1.556)	<.001	1.358(1.172-1.574)	<.001
Exposure: Fine-Gray	1.341(1.163-1.546)	<.001	1.354(1.175-1.561)	<.001
Stroke				
No-exposure	Reference			
Exposure: Cox	1.279 (1.022-1.601)	.03	1.284(1.026-1.608)	.03
Exposure: Fine-Gray	1.277(1.026-1.59)	.03	1.282(1.03-1.595)	.03

Abbreviation: HR, hazard ratio; CI, confidence interval; CVD, cardiovascular disease

a Childhood interparental violence exposure was defined as witnessing interparental violence sometimes or often when participants were aged before 17 years.

b HR was estimated using Fine-Gray sub-distribution models.

c Heart disease included heart attack, angina, coronary heart disease, heart failure, or other heart problems.

d Model 1: adjusted for age, and sex.

e Model 2: adjusted for age, sex, residence, education, smoking status, and current drinking.

© 2024 Cui C et al. *JAMA Network Open.*

eTable 4. Subgroup Associations of Interparental Physical Violence Experience and Incident Cardiovascular Disease

	CVD		Heart disease [b]		Stroke	
	HR (95% CI)	$P_{interaction}$	HR (95% CI)	$P_{interaction}$	HR (95% CI)	$P_{interaction}$
**Sex**		0.88		0.80		0.10
Men						
No-exposure	Reference					
Exposure [a]	1.372(1.124-1.674)		1.316(1.035-1.673)		1.044(0.795-1.371)	
None	Reference					
Rarely	1.014(0.854-1.205)		1.012(0.825-1.241)		1.428(1.015-2.008)	
Sometimes	1.347(1.081-1.679)		1.328(1.021-1.726)		2.127(1.164-3.888)	
Often	1.513(0.97-2.36)		1.272(0.717-2.255)		1.196(1.05-1.363)	
Women						
No-exposure	Reference					
Exposure [a]	1.351(1.14-1.602)		1.376(1.141-1.66)		1.056(0.753-1.482)	
None	Reference					
Rarely	1.156(0.974-1.374)		1.141(0.942-1.381)		1.125(0.82-1.544)	
Sometimes	1.299(1.063-1.587)		1.277(1.021-1.597)		1.051(0.709-1.558)	
Often	1.6(1.183-2.165)		1.762(1.278-2.431)		1.13(0.601-2.122)	
**Physical abuse by parent**		0.33		0.81		0.30
No-abuse						
No-exposure	Reference					
Exposure [a]	1.405(1.153-1.712)		1.35(1.077-1.692)		1.37(0.971-1.932)	
None	Reference					
Rarely	1.027(0.879-1.2)		1.034(0.866-1.234)		0.967(0.735-1.273)	
Sometimes	1.352(1.078-1.695)		1.256(0.966-1.634)		1.374(0.936-2.015)	
Often	1.622(1.1-2.392)		1.717(1.126-2.621)		1.329(0.629-2.806)	
Abuse exposure						
No-exposure	Reference					
Exposure [a]	1.242(1.037-1.487)		1.31(1.066-1.61)		1.098(0.804-1.499)	
None	Reference					
Rarely	1.122(0.914-1.377)		1.102(0.868-1.4)		1.217(0.878-1.688)	
Sometimes	1.207(0.982-1.484)		1.277(1.009-1.617)		1.032(0.717-1.486)	
Often	1.495(1.071-2.088)		1.543(1.052-2.262)		1.557(0.897-2.702)	
**Adult depression**		0.10		0.11		0.14
No depression						
No-exposure	Reference					
Exposure [a]	1.381(1.159-1.647)		1.419(1.163-1.732)		1.337(0.987-1.812)	
None	Reference					
Rarely	1.053(0.897-1.234)		1.072(0.893-1.286)		0.996(0.755-1.314)	

© 2024 Cui C et al. *JAMA Network Open.*

Sometimes	1.36(1.114-1.659)	1.404(1.12-1.758)	1.314(0.934-1.85)
Often	1.507(1.054-2.154)	1.54(1.026-2.311)	1.424(0.759-2.671)
Depression			
No-exposure	Reference		
Exposure [a]	1.066(0.843-1.347)	1.046(0.802-1.364)	0.916(0.6-1.398)
None	Reference		
Rarely	1.142(0.921-1.416)	1.09(0.85-1.398)	1.252(0.886-1.769)
Sometimes	1.041(0.796-1.361)	0.983(0.722-1.338)	0.855(0.519-1.408)
Often	1.259(0.804-1.971)	1.338(0.822-2.178)	1.3(0.609-2.776)

Abbreviation: CVD, cardiovascular disease.
a Childhood interparental violence exposure was defined as witnessing interparental violence sometimes or often when participants were aged before 17 years.
b Heart disease included heart attack, angina, coronary heart disease, heart failure, or other heart problems.

Analyses were adjusted for age, sex, residence, education, smoking status, and current drinking, if not stratified.

© 2024 Cui C et al. *JAMA Network Open.*

eTable 5. Association of Interparental Physical Violence Experience and Adult Depressive Symptoms at Baseline, 2011

	Case (prevalence) of depressive symptoms [a]	OR (95% CI)	
		Model 1 [c]	Model 2 [d]
Dichotomous			
No-exposure	2112 (24.6%)		
Exposure [b]	259 (34.0%)	1.572(1.339-1.843)	1.542(1.31-1.812)
Frequency			
None	1766 (23.8%)		
Rarely	346 (29.7%)	1.465(1.274-1.683)	1.429(1.24-1.644)
Sometimes	204 (33.9%)	1.687(1.407-2.016)	1.642(1.367-1.968)
Often	55 (34.2%)	1.565(1.113-2.178)	1.555(1.101-2.174)
P for trend [e]		<.001	<.001

Abbreviation: OR, Odds ratio; CI, confidence interval

a Depressive symptoms is defined as total CES-D score of 12 or higher.

b Childhood interparental violence exposure was defined as witnessing interparental violence sometimes or often when participants were aged before 17 years.

c Model 1: adjusted for age, and sex.

d Model 2: adjusted for age, sex, residence, education, smoking status, and current drinking.

e P for trend was calculated using frequency of witnessing interparental violence ranging between none and often as a continuous variable.

© 2024 Cui C et al. *JAMA Network Open.*

eTable 6. Proportions of the Association Between Childhood Interparental Physical Violence Exposure and Cardiovascular Diseases Attributable to Adult Depressive Symptoms

	HR (95% CI)	PERM, %
CVD		
No-exposure	Reference	
Exposure, multivariate model	1.364(1.199-1.552)	
Exposure, depressive symptoms adjusted	1.256(1.092-1.446)	11.0
Heart disease		
No-exposure	Reference	
Exposure, multivariate model	1.358(1.172-1.574)	
Exposure, depressive symptoms adjusted	1.270(1.083-1.490)	11.1
Stroke		
No-exposure	Reference	
Exposure, multivariate model	1.284(1.026-1.608)	
Exposure, depressive symptoms adjusted	1.171(0.914-1.499)	15.6

Abbreviation: CVD, cardiovascular disease; PERM, percentage of excess risk mediated.

Heart disease included heart attack, angina, coronary heart disease, heart failure, or other heart problems.
Childhood interparental violence exposure was defined as witnessing interparental violence sometimes or often when participants were aged before 17 years.

Multivariate model: adjusted for age, sex, residence, education, smoking status, and current drinking.

© 2024 Cui C et al. *JAMA Network Open.*

eFigure. Inclusion and Exclusion Flowchart for Participants in the Study

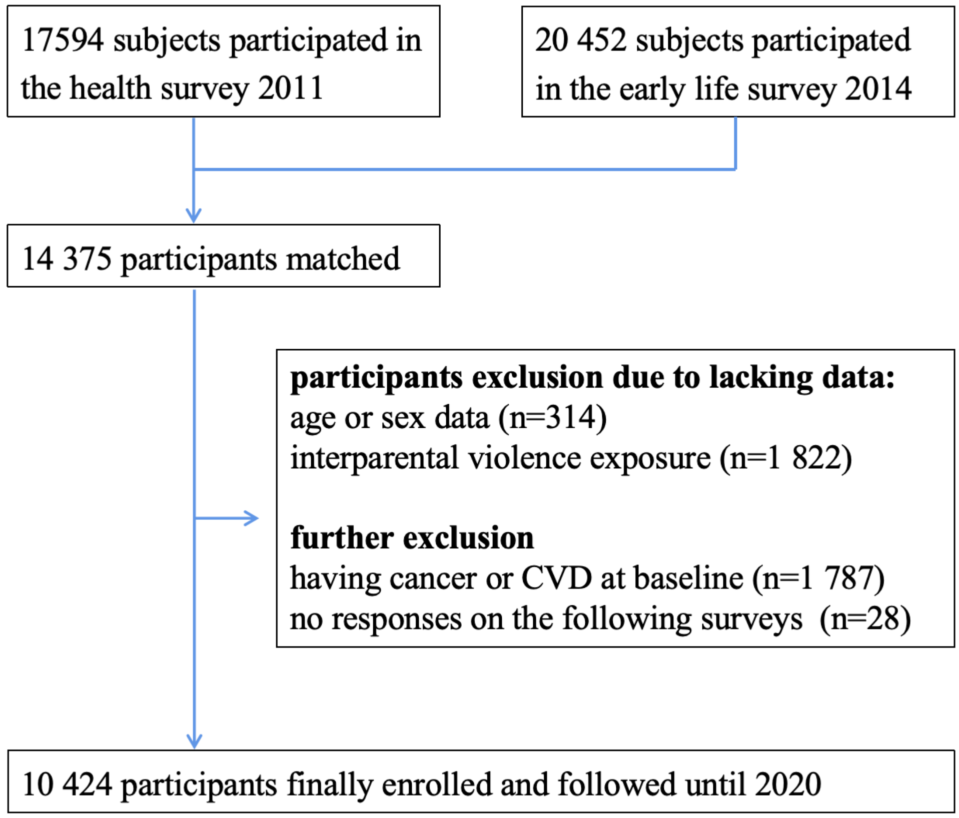

© 2024 Cui C et al. *JAMA Network Open.*
