# Supplementary material for: Childhood Exposure to Interparental Physical Violence and Adult Cardiovascular Disease
Source: JAMA Netw Open. 2024 Dec 20;7(12):e2451806. doi: 10.1001/jamanetworkopen.2024.51806 (PMC11662254; doi:10.1001/jamanetworkopen.2024.51806)
Supplement: Supplement 2. — Data Sharing Statement [file jamanetwopen-e2451806-s002.pdf]

# Data Sharing Statement

Cui. Childhood Exposure to Interparental Physical Violence and Adult Cardiovascular Disease. *JAMA Netw Open*. Published December 20, 2024. doi:10.1001/jamanetworkopen.2024.51806

**Data**
**Data available:** Yes
**Data types:** Deidentified participant data
**How to access data:** The final datasets generated for this study are available on reasonable request to the corresponding author (zhiyuanwu@hsph.harvard.edu).
**When available:** With publication

**Supporting Documents**
**Document types:** None

**Additional Information**
**Who can access the data:** Researchers whose proposed use of the data has been approved.
**Types of analyses:** for any purpose or for a specified purpose
**Mechanisms of data availability:** after approval of a proposal
